# Association between Phytochemical Index and Inflammation in Korean Adults

**DOI:** 10.3390/antiox11020348

**Published:** 2022-02-10

**Authors:** Chaehyun Kim, Kyong Park

**Affiliations:** Department of Food & Nutrition, Yeungnam University, 280 Daehak-ro, Gyeongsan 38541, Korea; chaehyunkim@yu.ac.kr

**Keywords:** inflammation, phytochemicals, C-reactive protein, white blood cells, dietary, Korean

## Abstract

While the relationship between phytochemicals and inflammation has been confirmed by in vivo or in vitro studies, large-scale epidemiological studies comprehensively analyzing phytochemical-rich food groups remain scarce. Therefore, we evaluated the association between the phytochemical index (PI) and the inflammation levels in Korean adults. The data were derived from the 2015–2018 Korea National Health and Nutrition Examination Survey, and a total of 18,699 participants were analyzed. High-sensitivity C-reactive protein (hs-CRP) levels >3.0 mg/L and white blood cell (WBC) counts >10 × 10^3^/μL were defined as “elevated.” The PI was calculated based on eight food groups using a 24-h dietary recall. The odds ratio (OR) and confidence interval (CI) for elevated hs-CRP levels and WBC counts, according to the PI quintile, were calculated using the multivariable logistic regression analysis. Participants in the higher PI group had lower means of hs-CRP levels and WBC counts than those in the lower PI group (all *p* for trend <0.001). In fully adjusted logistic regression models, elevated hs-CRP levels and WBC counts in the highest PI group were lower by 40% (OR: 0.61, 95% CI: 0.49–0.76) and 34% (OR: 0.66, CI: 0.47–0.93), respectively. Conclusions: A high consumption of phytochemical-rich foods is associated with lower inflammation. This suggests that adopting phytochemical-rich dietary patterns may be an effective approach for reducing inflammation.

## 1. Introduction

Inflammation is an immune response that is essential for recovery from infections and regeneration of damaged tissues in the body [1]. However, long-term persistence of a chronic inflammatory state can cause oxidative damage and lead to the development of chronic diseases [1], such as cardiovascular disease (heart attack and stroke), cancer, chronic respiratory disease (chronic obstructive pulmonary disease and asthma), digestive disease, and diabetes [2]. According to the Korea Centers for Disease Control and Prevention, deaths due to chronic diseases accounted for 80% of total deaths among Koreans in 2017, which was higher than the global average of 71% [3,4].

Inflammatory conditions in humans trigger the biosynthesis of high-sensitivity C-reactive protein (hs-CRP), an inflammatory marker, in the hepatocytes in response to inflammatory cytokines, such as interleukin-6; hs-CRP is then secreted into the bloodstream [5]. Hs-CRP is widely used as a biochemical indicator for the diagnosis or early detection of chronic diseases owing to advantages such as its high sensitivity, rapid reaction rate, high precision in measuring both high and low concentrations, and indirect prediction of chronic disease risk [6]. White blood cell (WBC) proliferation is caused by various inflammatory mediators, such as cytokines and chemokines [7]. The WBC count is used as an inflammatory biomarker because it is involved in the development of numerous inflammatory responses that occur with foreign agent invasion, such as following bacterial and viral infections and tumor immunity tissue damage [7].

Phytochemicals are non-nutritive chemicals produced as secondary products in plants and play several beneficial roles in humans, such as relieving oxidative stress, inhibiting inflammatory transcription factors, and regulating enzyme activity [8,9]. They serve as anti-inflammatory agents and antioxidants in the human body and regulate signaling pathways related to immune responses, resulting in reduced inflammation levels [9,10,11,12]. However, the effects of phytochemicals on inflammation may differ depending on ethnicity or food source type [12,13,14,15,16,17]. For example, a cross-sectional analysis of 5013 US men and women aged 30–75 years from the 1989–1990 Nurses’ Health Study and the 1993–1995 Health Professionals Follow-Up Study showed that nut consumption was inversely associated with hs-CRP levels [13]. In contrast, a cohort study of 783 Italian men and women aged ≥65 years from the 1998–2009 Invecchiare in Chianti Study demonstrated no association between resveratrol consumption and hs-CRP levels [15]. A recent randomized cross-over study of individuals with obesity or abdominal obesity aged 20–65 years, who underwent two eight-week interventions, found that a whole-grain-rich diet reduced the hs-CRP [16]. The majority of prior epidemiological studies that have investigated the association between phytochemicals and hs-CRP have been conducted in western countries and focused on a single food item or compound. Limited data is available in the Asian population, including Koreans [14], especially for comprehensively evaluated health effects of dietary phytochemicals in large epidemiologic study designs. 

Therefore, the current study aimed to analyze the association between phytochemical-rich food consumption and inflammation among Koreans using the phytochemical index (PI), which is a dietary index based on data from the Korea National Health and Nutrition Examination Survey (KNHANES).

## 2. Materials and Methods

### 2.1. Study Population

The KNHANES is a nationwide representative cross-sectional survey, which measures health behaviors, chronic disease prevalence, and food and nutritional status in the generally healthy Korean population. The KNHANES was first performed in 1998 and was conducted as a short-term survey once every three years. In 2007, the KNHANES was redesigned into a rolling sampling survey and has since been conducted every year. Its target population includes nationally representative civilians aged ≥1 year living in South Korea. A detailed description of this survey is reported elsewhere [18]. The present study analyzed data from the KNHANES 2015–2018 in which hs-CRP levels and WBC counts were measured.

Overall, 31,649 individuals participated in the 2015–2018 KNHANES. Exclusion criteria comprised the following: (1) <19 years of age (*n* = 6315); (2) missing hs-CRP levels or WBC counts; inflammation biomarker levels exceeding the measurable range; or suspected acute infection, systemic inflammation, or tissue damage (*n* = 3477) [18,19]; and (3) extremely low (<500 kcal) or high (>5000 kcal) total daily energy intake (*n* = 3158) [20]. Finally, 18,699 participants were included in the main analysis.

### 2.2. Assessment of Demographic and Lifestyle Information

Data on the participants’ health behaviors, such as alcohol consumption and smoking status, were collected through self-reported questionnaires while all other data such as education level and physical activity level, were collected through interviews conducted by a trained investigator [18]. The participants’ body mass index (BMI) was calculated by dividing the individuals’ weight (kg) by the square of their height (m^2^). Their educational level was classified into three categories: middle school graduates or below, high school graduates, and college graduates or above. Participants were classified as non-smokers, former smokers, or current smokers. The daily alcohol consumption (servings/day) was calculated by multiplying the frequency of drinking for the past year (from the time of the survey) by the amount of alcohol consumed on a single occasion. Metabolic equivalent tasks (METs-h/week) were calculated based on the number of days and hours of intense, moderate, or mild physical activity level by assigning a weighted value to each exercise intensity [21].

### 2.3. Measurement of hs-CRP Levels and WBC Counts

Blood samples were collected in a 3-mL tube containing ethylenediamine tetra-acetic acid; the contents were mixed for 10 min using a roller mixer to prevent coagulation [22]. Hs-CRP levels were measured using the Cobas (Roche, Mannheim, Germany) instrument and quantified using the immunoturbidimetric method [18]. Elevated hs-CRP levels were defined as >3.0 mg/L according to the American Heart Association and the Centers for Disease Control and Prevention of the US [23]. The WBC count was measured by flow cytometry using a semiconductor laser, XN-9000 equipment (Sysmex, Tokyo, Japan) [18], and an elevated WBC count was defined as >10 × 10^3^/μL [24].

### 2.4. Nutrition Survey Data and PI

The KNHANES investigated participants’ food intake and contents one day before the nutrition survey, using the 24-h dietary recall method [25]. To ensure accurate assessments, each trained nutrition survey team (consisting of two dieticians) visited the homes of the survey participants and conducted individual interviews using supplementary materials, such as measuring cups and measuring spoons [25].

The Korean version of the PI was calculated based on a previous study [26]. The PI was defined as the percentage of daily energy intake derived from various phytochemical-rich foods divided by the total energy intake [27]; the Korean version of the PI was computed in consideration of Korean dietary habits [26]. For example, seaweed is frequently consumed among Koreans [28]; thus, it is included in the PI calculation. Therefore, the following eight food groups were finally included in the PI calculation: whole grains and whole-grain products, vegetables, fruits, legumes, soybeans and soybean products, nuts and seeds, olives and olive oil, and seaweed [27,28].

### 2.5. Statistical Analysis

As the KNHANES used a complex sampling design, the statistical analysis in this study was performed by reflecting all stratification variables, clustering variables, and weights. The participants’ characteristics were presented as frequencies and percentages for categorical variables and as means and standard errors for continuous variables. The multivariable linear regression analysis was used to examine the adjusted mean of the hs-CRP and WBC count by the PI quintile. The multivariable logistic regression analysis was used to calculate the odds ratios (ORs) and 95% confidence intervals (CIs) for the association between the PI quintile and the elevated inflammation levels (hs-CRP levels (>3 mg/L) and WBC counts (>10 × 10^3^/μL). Multivariable linear regression analysis, using the median value of each quintile as a continuous variable, was performed to calculate the *p* for trend. Potential confounding variables were selected through a comprehensive literature review and the preliminary analysis [29,30,31,32]. Consequently, four models were built as follows: Model 1 was an unadjusted; Model 2 was adjusted for age; Model 3 was adjusted for age, sex, BMI, education level, and physical activity level; and Model 4 included all variables of Model 3 plus alcohol consumption, smoking status, comparison with normal meals, meat and meat products, sweets, and total energy intake. Restricted cubic spline regression analysis with full adjustment was performed to analyze a dose−response relationship between the PI and the elevated hs-CRP levels/WBC counts. All statistical analyses were performed using the Statistical Analysis System (SAS; ver. 9.4, SAS Institute, Cary, NC, USA). Statistical significance was set at α = 0.05.

## 3. Results

### 3.1. Characteristics of the Participants

The general characteristics of the participants, according to the PI quintile, are shown in Table 1. The median values for the PI quintiles were 3.25, 8.03, 13.40, 20.25, and 32.73. PI values tended to be higher with older age (*p* < 0.001), female sex (*p* < 0.001), non-smoking status (*p* < 0.001), and higher physical activity level (*p* = 0.006) whereas lower values were seen with frequent alcohol consumption (*p* < 0.001) and higher intake of meat and meat products (*p* < 0.001), sweets (*p* < 0.001), and total energy (*p* < 0.001).

### 3.2. Association between PI and Inflammation Markers

The crude and adjusted means of hs-CRP levels and WBC counts, according to the PI quintile, are shown in Table 2. Both hs-CRP levels and WBC counts tended to be lower with a higher PI quintile in all statistical models, which showed significant linear relationships (all *p* for trend <0.001).

ORs and 95% CIs for elevated hs-CRP levels and WBC counts, according to the PI quintile, are shown in Table 3. In the unadjusted model, the PI was associated with 36% and 53% lower odds of elevated hs-CRP levels and WBC counts, respectively, in the group with the highest PI compared to those in the group with the lowest PI (hs-CRP (OR: 0.64, 95% CI: 0.53–0.77), WBC count (OR: 0.47, 95% CI: 0.34–0.64)); this indicated inverse associations between the PI and the odds of elevated hs-CRP levels/WBC counts (all *p* for trend <0.001). Similarly, Model 2 and Model 3, with adjusted covariates using the step-by-step approach, showed 45% (OR: 0.55, CI: 0.44–0.67) and 41% (OR: 0.59, CI: 0.47–0.74) lower odds of elevated hs-CRP levels, and 49% (OR: 0.51, CI: 0.37–0.72) and 32% (OR: 0.68, CI: 0.48–0.96) lower odds of elevated WBC counts, respectively. An inverse association was also found with the fully adjusted models. The ORs for elevated hs-CRP levels and WBC counts were significantly lower in the group with the highest PI (hs-CRP (OR: 0.61, 95% CI: 0.49–0.76, *p* for trend < 0.001), WBC count (OR: 0.66, CI: 0.47–0.93, *p* for trend = 0.03)).

**Figure 1:**
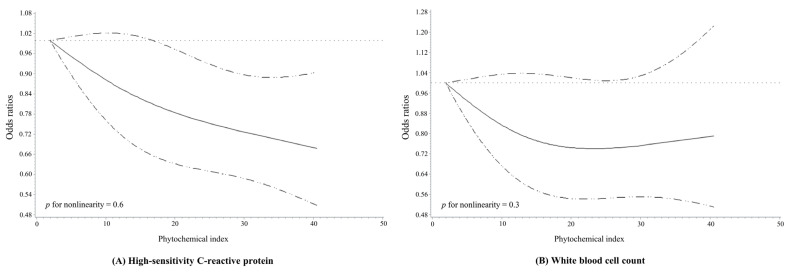
Multivariable adjusted odds ratios (95% confidence intervals) for the non-linear relationship of the phytochemical index with (**A**) high-sensitivity C-reactive protein levels of >3 mg/L and (**B**) white blood cell counts of >10 × 10^3^/μL. The model was adjusted for age, sex, body mass index, education level, physical activity level, smoking status, and alcohol consumption as well as a comparison with normal meals, meat and meat products, sweets, and total energy intake.

Spline curves analyzing the ORs and 95% CIs for the dose–response relationship between the PI and the elevated hs-CRP levels/WBC counts are presented in Figure 1. When all covariates were adjusted and three knots were randomly assigned and connected, the results showed that the PI was inversely associated with the odds of elevated hs-CRP levels (*p* for nonlinearity = 0.6) and WBC counts (*p* for nonlinearity = 0.3) in a dose–response manner.

### 3.3. Subgroup Analysis of PI and Elevated Inflammation Markers

**Figure 2:**
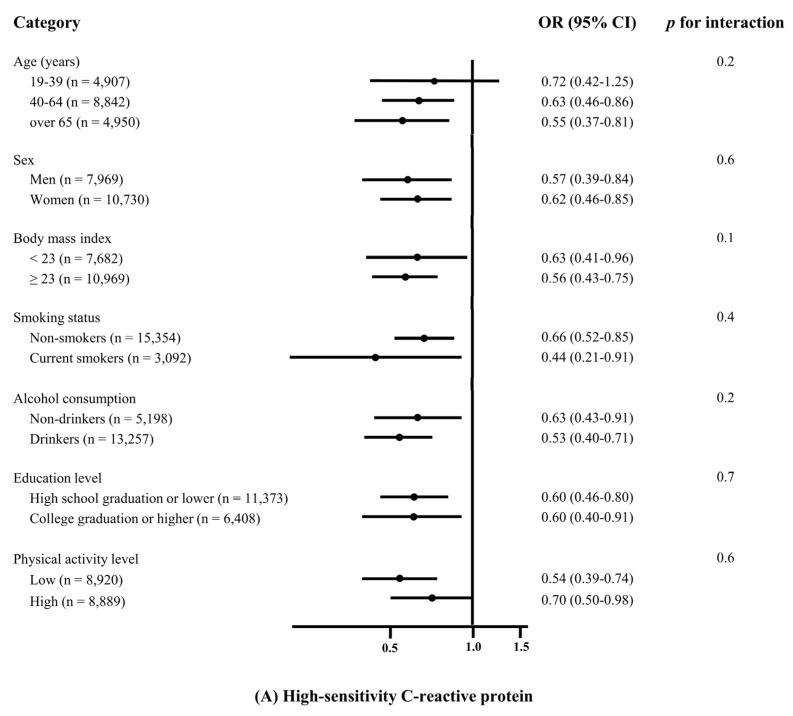

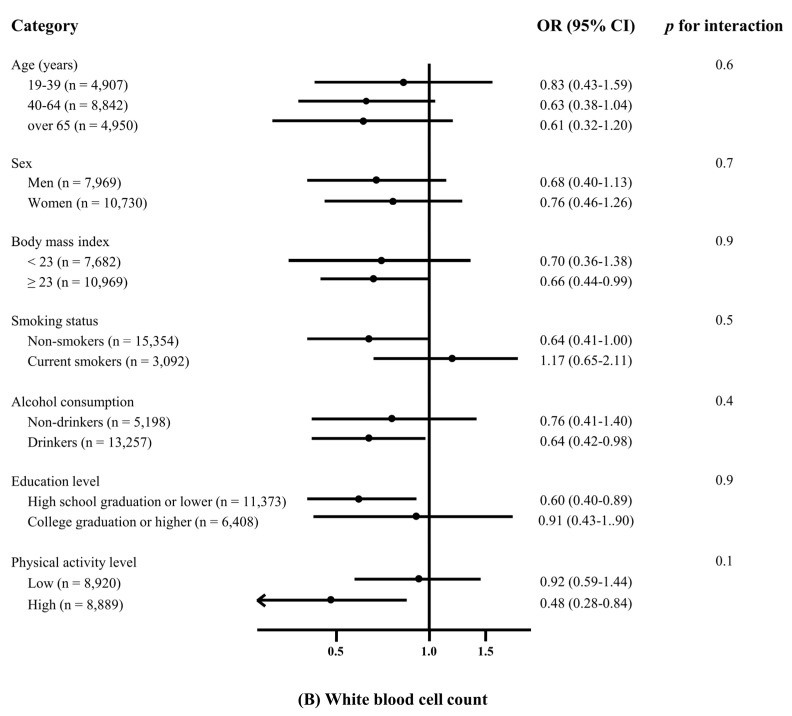
Effect of various demographic factors on the associations of the phytochemical index with (**A**) high-sensitivity C-reactive protein (>3 mg/L) and (**B**) white blood cells (>10 × 10^3^/μL). Odds ratios (ORs) and 95% confidence intervals (CIs) of high-sensitivity C-reactive protein and white blood cell count in the quintile of the phytochemical index were compared to the first quintile based on age, sex, body mass index, smoking status, alcohol consumption, education level, and physical activity level. The values were simultaneously adjusted for the listed variables and other potential confounders, which included comparisons to normal meals, meat and meat products, sweets, and total energy intake.

The association between the PI and the elevated inflammation markers in the subgroup analysis stratified by age, sex, BMI, smoking status, alcohol consumption, education level, and physical activity level are shown in Figure 2. No significant effect modification on the association between the PI and the hs-CRP levels/WBC counts was found (all *p* for interaction >0.05).

## 4. Discussion

This study investigated the association between the PI and the inflammation levels in Korean adults aged <19 years from the 2015–2018 KNHANES. A higher PI was inversely associated with the odds of elevated hs-CRP levels and WBC counts. The analysis of the dose–response relationship revealed an inverse linear association between the PI and the inflammation markers.

The results of previous epidemiological studies and clinical trials that have analyzed the association between phytochemical-rich plant food intake and inflammation levels were consistent with the results of the present study [13,16,33]. For example, a previous study evaluated the longitudinal association between lycopene intake and hs-CRP levels in 23,935 US men and women aged ≥20 years for 76.4 months, using data from the two-year National Health and Nutrition Examination Survey cycles (1999–2010) in the US [33]. It was shown that a higher amount of lycopene consumption was associated with lower hs-CRP levels [33]. The positive effects of a large intake of phytochemical-rich plant foods on hs-CRP levels can be explained by the action of polyphenols (a type of phytochemical), which protects cells by inhibiting pro-inflammatory enzymes (e.g., cyclooxygenase-2, lysyl oxidase, and inducible nitric oxide synthase) and by activating nuclear factor erythroid 2-related factor 2 (a transcription factor) [34,35]. In addition, polyphenols remove reactive oxygen species and free radicals as well as protect cells from oxidative stress and inflammation, thereby suppressing hs-CRP levels [34,36,37]. Flavonoids also remove trace elements involved in the production of reactive oxygen species; inhibit the function of microsomal mono-oxidase, glutathione-S-transferase, mitochondrial succinate, and nicotinamide adenine dinucleotide oxidase; and they also bind to metal ions to exert strong antioxidant effects [38,39,40]. Carotenoids display antioxidant effects and inhibit the production of hs-CRP as follows: transfer electrons to peroxyl radicals to produce ROO^−^, release allylic hydrogens to produce ROOH, and add ROO to double bonds to form ROOCAR^•^ [41]. Moreover, a recent study reported that lutein inhibits the production of hs-CRP by blocking the neural pathway of the nuclear factor kappa-light-chain-enhancer of activated B cells in Mueller cells, which are a primary source of inflammatory cytokines [36,42]. Lutein is a hydroxycarotenoid characterized by a hydroxyl group located at either end of the molecule; it removes singlet oxygen and lowers hs-CRP levels [42,43]. However, the studies that analyzed the association with phytochemicals using WBC counts were very limited. Therefore, current evidence indicates that phytochemicals have important antioxidant and anti-inflammatory effects, which result in the reduction of hs-CRP levels [5,38].

Plant foods, such as whole grains, vegetables, fruits, nuts, and legumes, are rich in phytochemicals (non-nutrient substances), along with nutrients essential for the body [44]. The consumption of phytochemical-rich plant foods offers many health benefits [45]. Wheat grains contain various nutrients and physiologically active substances, including powerful antioxidants (phenolic acids, flavonoids, carotenoids, vitamin E, and phytosterols) as well as water-soluble β-glucan [46,47,48]. Vegetables and fruits contain large amounts of vitamins and minerals, dietary fibers, carotenoids, and flavonoids [49]. Legumes, such as soybeans, are known for being plant-based protein sources; they contain large amounts of phytochemicals, such as isoflavones, saponins, and phytosterols as well as soluble and insoluble fibers, thereby offering various micronutrients to consumers [50]. Nuts are rich in phytochemicals, including polyphenols, ellagitannins, and proanthocyanidins, which are known to have anti-inflammatory and antioxidant properties, promote detoxification, and reduce low-density lipoprotein cholesterol [51,52,53]. Nuts are also rich in omega-3 fatty acids and vitamin E, which help protect neurons from free radicals, such as reactive oxygen and nitrogen species, and aid in the repair of damaged cells [53]. Phytosterols, such as β-sitosterol and fucosterol, are found in seaweed, which is also rich in vitamin B12, making it an excellent source of nutrition that is often deficient in general plant foods [54,55]. Olives and olive oil are also rich in phenolic phytochemicals, including hydroxytyrosol and oleuropein, which activate endogenous antioxidant systems in the body and remove free radicals, showing anticancer, antiangiogenic, and anti-inflammatory properties [56]. A balanced diet of various plant foods provides several types of phytochemicals, and the combination of vitamins, minerals, and non-nutrients may offer synergistic benefits [57].

This study has several limitations that should be considered. First, as the KNHANES was a cross-sectional survey, there may be a possibility of reverse causality. To minimize this issue, individuals whose inflammation levels were considered to be associated with acute infection, systemic inflammation, or tissue damage were excluded at the time of the survey, and data analysis was performed after adjusting for various potential confounding factors. Nevertheless, there may be still a possibility that residual confounding factors remained. Second, information on diet and nutrient intake was obtained using a 24-h dietary recall method; as a result, the participants’ usual dietary habits may not have been accurately reflected. Therefore, our analysis accounted for whether the determined dietary intake levels were below or above the usual intake levels. Moreover, participants with extremely low or high energy intake levels were excluded from the analysis. Third, as the PI was an exposure factor and calculated using calories, foods rich in phytochemicals but without calories were not taken into account. Fourth, the elevated hs-CRP level used as the reference value in the present study was based on the American population. Therefore, we were unable to apply an appropriate standard tailored for the Korean ethnicity and dietary habits.

However, despite these limitations, this study is significant in that, to the best of our knowledge, it is the first to analyze the association between the PI and the inflammation levels in Korean adults. Furthermore, this study used WBC counts as an additional inflammatory marker to CRP to confirm the results that an inverse association occurred with phytochemical-rich plant food consumption. Our findings can be used to inform the development of strategies and dietary guidelines aimed at the reduction of chronic inflammation in Korean adults with chronic diseases.

## 5. Conclusions

This study confirmed that there was a significant inverse association between phytochemical-rich plant food consumption and elevated inflammation markers, hs-CRP levels (>3.0 mg/L) and WBC counts (>10 × 10^3^/μL). This inverse linear association also showed a dose–response relationship. Further studies are needed to establish a reference value for inflammatory biomarkers that is tailored to the Korean population. Large-scale clinical trials and prospective cohort studies are required to clearly determine the effects of phytochemicals on inflammation.

## Figures and Tables

**Table 1:** General characteristics of the study participants, according to the PI quintiles (KNHANES 2015–2018, *n* = 18,699).

	Quintile of PI					*p*-Value
	Q1	Q2	Q3	Q4	Q5	
Number of participants	3739	3740	3740	3740	3740	
PI, median (range)	3.25 (0.00–5.69)	8.03 (5.69–10.62)	13.40 (10.62–16.59)	20.25 (16.59–25.11)	32.73 (25.11–98.21)	
Age (years)	43.88 ± 0.28	48.52 ± 0.27	52.87 ± 0.26	56.05 ± 0.25	58.46 ± 0.23	<0.001
Sex						<0.001
Men	1992 (53.28)	1868 (49.95)	1529 (40.88)	1420 (37.97)	1160 (31.02)	
Women	1747 (46.72)	1872 (50.05)	2211 (59.12)	2320 (62.03)	2580 (68.98)	
Education level						<0.001
Middle school graduation or lower	805 (22.71)	940 (26.50)	1209 (33.94)	1322 (37.25)	1420 (39.68)	
High school graduation	1330 (37.53)	1175 (33.13)	1045 (29.34)	1085 (30.57)	1042 (29.11)	
College graduation or higher	1409 (39.76)	1432 (40.37)	1308 (36.72)	1142 (32.18)	1117 (31.21)	
Smoking status						<0.001
Non-smokers	1844 (49.96)	2052 (55.47)	2340 (63.52)	2454 (66.68)	2656 (71.94)	
Former smokers	775 (21.00)	876 (23.68)	795 (21.58)	807 (21.93)	755 (20.45)	
Current Smokers	1072 (29.04)	771 (20.85)	549 (14.90)	419 (11.39)	281 (7.61)	
Body mass index (kg/m^2^)	24.07 ± 0.06	24.04 ± 0.06	24.04 ± 0.06	24.00 ± 0.06	23.92 ± 0.05	0.359
Alcohol consumption	1.19 ± 0.03	1.02 ± 0.03	0.68 ± 0.02	0.54 ± 0.02	0.36 ± 0.02	<0.001
Physical activity level ^1^	17.10 ± 0.40	16.34 ± 0.37	16.56 ± 0.38	17.68 ± 0.40	18.09 ± 0.36	0.006
Meat and meat products intake	279.36 ± 6.00	244.35 ± 4.94	184.18 ± 3.90	146.47 ± 3.29	88.61 ± 2.23	<0.001
Sweets intake	39.09 ± 1.21	39.88 ± 1.09	36.41 ± 1.08	32.34 ± 0.98	27.83 ± 0.86	<0.001
Total energy intake (kcal/day)	2078.69 ± 15.06	2059.29 ± 13.57	1931.02 ± 12.44	1859.52 ± 11.72	1724.84 ± 10.99	<0.001

PI, phytochemical index; KNHANES, Korea National Health and Nutrition Examination Survey; Q, quintile. Values are presented as mean ± standard error or as *n* (%). ^1^ Physical activity level was calculated as metabolic equivalent task-hours per week (METs-h/week).

**Table 2:** Crude and adjusted means of hs-CRP levels and WBC counts, according to the PI quintile.

	Quintile of PI					*p* for Trend
	Q1	Q2	Q3	Q4	Q5	
Hs-CRP (mg/L)						
Model 1	1.10 ± 0.03	1.00 ± 0.02	1.00 ± 0.02	1.05 ± 0.03	0.95 ± 0.02	<0.001
Model 2	1.18 ± 0.03	1.05 ± 0.02	1.02 ± 0.02	1.05 ± 0.03	0.93 ± 0.02	<0.001
Model 3	1.15 ± 0.03	1.04 ± 0.02	1.01 ± 0.02	1.07 ± 0.03	0.96 ± 0.02	<0.001
Model 4	1.14 ± 0.04	1.03 ± 0.04	0.98 ± 0.04	1.04 ± 0.04	0.93 ± 0.04	<0.001
WBC count (10^3^/μL)						
Model 1	6.68 ± 0.04	6.42 ± 0.03	6.28 ± 0.03	6.23 ± 0.03	6.05 ± 0.03	<0.001
Model 2	6.61 ± 0.04	6.38 ± 0.03	6.26 ± 0.03	6.23 ± 0.03	6.07 ± 0.03	<0.001
Model 3	6.51 ± 0.04	6.33 ± 0.03	6.27 ± 0.03	6.29 ± 0.03	6.19 ± 0.03	<0.001
Model 4	6.62 ± 0.06	6.50 ± 0.06	6.44 ± 0.06	6.49 ± 0.06	6.39 ± 0.06	<0.001

Values are mean ± standard error. Q, quintile; PI, phytochemical index; hs-CRP, high-sensitivity C-reactive protein; WBC, white blood cells. Model 1: unadjusted. Model 2: adjusted for age (continuous). Model 3: Model 2 plus additional adjustments for sex, body mass index (continuous), education level (middle school graduation or lower, high school graduation, and college graduation or higher), and physical activity level (continuous). Model 4: Model 3 plus additional adjustments for alcohol consumption (continuous), smoking status (non-smokers, former smokers, and current smokers), comparison with normal meals (low, moderate, and high), meat and meat products (continuous), sweets (continuous), and total energy intake (continuous).

**Table 3:** Odds ratios (95% confidence intervals) for the hs-CRP levels of >3 mg/L and WBC counts of >10 × 10^3^/μL, according to PI quintile.

	Quintile of PI					*p* for Trend
	Q1	Q2	Q3	Q4	Q5	
	(*n* = 3739)	(*n* = 3740)	(*n* = 3740)	(*n* = 3740)	(*n* = 3740)	
Hs-CRP						
Cases (%)	338 (9.04)	265 (7.09)	255 (6.82)	277 (7.41)	219 (5.86)	
Model 1	Ref	0.78 (0.64–0.96)	0.77 (0.63–0.94)	0.88 (0.73–1.06)	0.64 (0.53–0.77)	<0.001
Model 2	Ref	0.75 (0.61–0.91)	0.70 (0.58–0.86)	0.78 (0.63–0.95)	0.55 (0.44–0.67)	<0.001
Model 3	Ref	0.79 (0.65–0.97)	0.71 (0.58–0.88)	0.83 (0.67–1.03)	0.59 (0.47–0.74)	0.001
Model 4	Ref	0.81 (0.66–0.99)	0.72 (0.59–0.89)	0.85 (0.69–1.06)	0.61 (0.49–0.76)	<0.001
WBC count						
Cases (%)	179 (4.79)	121 (3.24)	99 (2.65)	106 (2.83)	93 (2.49)	
Model 1	Ref	0.62 (0.47–0.82)	0.58 (0.46–0.76)	0.55 (0.42–0.73)	0.47 (0.34–0.64)	<0.001
Model 2	Ref	0.63 (0.48–0.84)	0.61 (0.46–0.82)	0.59 (0.45–0.79)	0.51 (0.37–0.72)	<0.001
Model 3	Ref	0.70 (0.53–0.93)	0.65 (0.50–0.86)	0.72 (0.54–0.97)	0.68 (0.48–0.96)	0.049
Model 4	Ref	0.70 (0.53–0.93)	0.69 (0.51–0.92)	0.71 (0.53–0.95)	0.66 (0.47–0.93)	0.03

Q, quintile; PI, phytochemical index; hs-CRP, high-sensitivity C-reactive protein; WBC, white blood cell; Ref, reference. Model 1: unadjusted. Model 2: adjusted for age (continuous). Model 3: Model 2 plus additional adjustment for sex, body mass index (continuous), education level (middle school graduation or lower, high school graduation, and college graduation or higher), and physical activity level (continuous). Model 4: Model 3 plus additional adjustment for alcohol consumption (continuous), smoking status (non-smokers, former smokers, and current smokers), comparison with normal meals (low, moderate, and high), meat and meat products (continuous), sweets (continuous), and total energy intake (continuous).

## Data Availability

The datasets supporting the conclusions of this article are available from the Korea Centers for Disease Control and Prevention on reasonable request. These datasets are available with a permission at the following URLs: https://knhanes.kdca.go.kr/knhanes/sub03/sub03_02_05.do (accessed on 3 January 2022).
